# Influenza surveillance with Baidu index and attention-based long short-term memory model

**DOI:** 10.1371/journal.pone.0280834

**Published:** 2023-01-23

**Authors:** Shangfang Dai, Litao Han

**Affiliations:** 1 School of Economics and Management, Tsinghua University, Beijing, China; 2 School of Mathematics, Renmin University of China, Beijing, China; University of Kurdistan Hewler, IRAQ

## Abstract

**Background:**

The prediction and prevention of influenza is a public health issue of great concern, and the study of timely acquisition of influenza transmission trend has become an important research topic. For achieving more quicker and accurate detection and prediction, the data recorded on the Internet, especially on the search engine from Google or Baidu are widely introduced into this field. Moreover, with the development of intelligent technology and machine learning algorithm, many updated and advanced trend tracking and forecasting methods are also being used in this research problem.

**Methods:**

In this paper, a new recurrent neural network architecture, attention-based long short-term memory model is proposed for influenza surveillance. This is a kind of deep learning model which is trained by processing from Baidu Index series so as to fit the real influenza survey time series. Previous studies on influenza surveillance by Baidu Index mostly used traditional autoregressive moving average model or classical machine learning models such as logarithmic linear regression, support vector regression or multi-layer perception model to fit influenza like illness data, which less considered the deep learning structure. Meanwhile, some new model that considered the deep learning structure did not take into account the application of Baidu index data. This study considers introducing the recurrent neural network with long short-term memory combined with attention mechanism into the influenza surveillance research model, which not only fits the research problems well in model structure, but also provides research methods based on Baidu index.

**Results:**

The actual survey data and Baidu Index data are used to train and test the proposed attention-based long short-term memory model and the other comparison models, so as to iterate the value of the model parameters, and to describe and predict the influenza epidemic situation. The experimental results show that our proposed model has better performance in the mean absolute error, mean absolute percentage error, index of agreement and other indicators than the other comparison models.

**Conclusion:**

Our proposed attention-based long short-term memory model vividly verifies the ability of this attention-based long short-term memory structure for better surveillance and prediction the trend of influenza. In comparison with some of the latest models and methods in this research field, the model we proposed is also excellent in effect, even more lightweight and robust. Future research direction can consider fusing multimodal data based on this model and developing more application scenarios.

## Introduction

Seasonal influenza has the ability of transmission among the crowds, which is a serious public health problem that causes acute respiratory infectious diseases in the infected, and has a great impact on life health and social activities. According to the report from the World Health Organization (WHO), the annual influenza epidemics are estimated to result in about 3 to 5 million cases of severe illness, and about 290 000 to 650 000 respiratory deaths worldwide [1]. However, the traditional monitoring systems that use both virologic and clinical data are usually published or reported on a weekly basis, which will lag behind the latest progress of influenza epidemic [2], including the systems employed by the U.S. Centers for Disease Control and Prevention (CDC), the European Influenza Surveillance Scheme (EISS), and the Chinese National Influenza Center (CNIC) [3]. Taking CNIC as an example, the released influenza like case data often has a delay of one to two weeks. If we can analyse and predict the spread of influenza to give early warning in time, and find out the progress of the disease as early as possible and take countermeasures, we can effectively reduce the harm of such health problems. Therefore, more timely and accurate monitoring of influenza epidemic has been a research hotspot.

In the information age, people’s search behaviour on the Internet reflects a lot of information in daily life, including the search behaviour related to the prevention, infection, symptoms and treatment of influenza diseases. Through the analysis and mining of internet search data, many studies have been done to monitor and predict the epidemic dynamics of influenza in time. The most famous research done by Ginsberg et al. in 2009 [2], used the search records of Google search engine to monitor and predict the influenza epidemic trend. This paper also designed the influenza prediction system Google Flu Trends (GTF), which has been put into use for many years. The model has achieved good results in the practical application in the following years. Based on the GTF system, many studies combined various statistical methods, machine learning methods and multi-index or cross-regional influenza data, which achieved rich experimental results [4–9].

Due to the Chinese Internet environment, the Google’s services are limited to some extent in Chinese Mainland, meanwhile, Baidu is the most widely used search engine in China in recent years, whose search engine index service is also the most recognized in China. Hence it is more reliable to use Baidu Index as the data source of influenza monitoring rather than directly apply the results of data modelling based on Google to model the flu trends in China. Most of the Chinese domestic researches draw on the corresponding research ideas and methods by using Baidu search data to predict the influenza virus epidemic in China with different algorithms, whose tools and application scenarios are as rich as the GFT researches [10–14]. Among them, the Chinese web search terms for Baidu quires used in this research field of influenza surveillance, are carefully tested and mutual enriched, which can give us a reliable reference for our model [15, 16].

By using Baidu Index data to establish the illness prediction system including the influenza surveillance system, the traditional time series models and machine learning models have been typically used. To make it more specific, the support vector machine algorithm is used by Liang et al. [10], Wang et al. [15] and Bu et al. [16], the autoregressive moving average model and its similar forms were established in [13, 17], and the regression models like linear regression and least absolute shrinkage and selection operator regression (LASSO regression) were used in many researches such as [12, 15, 16]. Almost all these existing research models have been tested and compared with our proposed model.

At present, there are few researches on the means of influenza monitoring through deep learning, especially in this field combined with Baidu Index. As a kind of neural network for modelling sequence data, the recurrent neural network (RNN) can "memory" the characteristic of time series, which considers the influence of the former sequence data on the latter, and solves the problem in the long-term relationship of time series [18]. Without considering the Baidu Index data source, some RNN-based deep learning method have already used in the influenza monitoring field, the model composed by genetic algorithm and long short-term memory structure, and its advanced version named GA ConvLSTM CNN composed by genetic algorithm, convolutional long short-term memory (LSTM) network and convolutional neural network (CNN) structure is done on ILI and weather data are proposed by Kara [19, 20]. There is also an attention-based RNN develop only on ILI data developed by Zhu et al. [21].

However, the epidemic infection data is characterized by that, on the one hand, it is an autocorrelation and periodic time series; on the other hand, from the principle of epidemic transmission, to a great extent, only a certain infection case data at the end of the time series can lead to subsequent infection case data. For influenza, that is to say, only the most recent cases of infectious diseases will be infectious, leading to infection cases in the next period of time, while earlier cases of infection have been cured and are almost no longer infectious. Most of the previously mentioned algorithm models can hardly take these two characteristics into account at the same time, moreover, although those works focusing on deep learning structure may have solved these two points through the complex network structure, they have almost no given consideration on the use of Baidu Index data. Thus, a research gap has formed, which provides a theoretical basis for us to turn to new deep learning algorithms. Hence, considering the feasibility of using search engine data for influenza monitoring [22] and the advantages of RNN-kind models in time series data processing, based on Baidu Index data and real influenza statistics, our study explores supervised machine learning experiments by using less data to get excellent fitting results.

In the next sections, a deep learning architecture is assembled to apply to this kind of research for the first time, which means, a new idea of influenza surveillance based on search engine data has been opened up. In Methods section, we will describe our data sources and models used in this study. The data we use are into influenza like case data and Baidu Index data respectively, which are joined together in helping to design a more efficient and robust model. In the model phase, the LSTM network built on attention-based architecture is established, the other models were taken for comparison, and the evaluation indexes that we will apply in the model experiment stage are also described. Our proposed deep learning model is considered to better capture and remember the characteristics of sequences by the function of LSTM cells and better focus on the data which may be more likely to cause new infections by the function of attention mechanism, which supports it to more perfectly fill the research gap pointed out before and achieve state-of-art results. In the Results section, we will do experiments for our proposed model and the most existing models involved in this research field, then take them into comparison. Finally, by analysing our experimental results, we will take several discussions on the Discussion section.

## Methods

### Methodology

**Fig 1:**
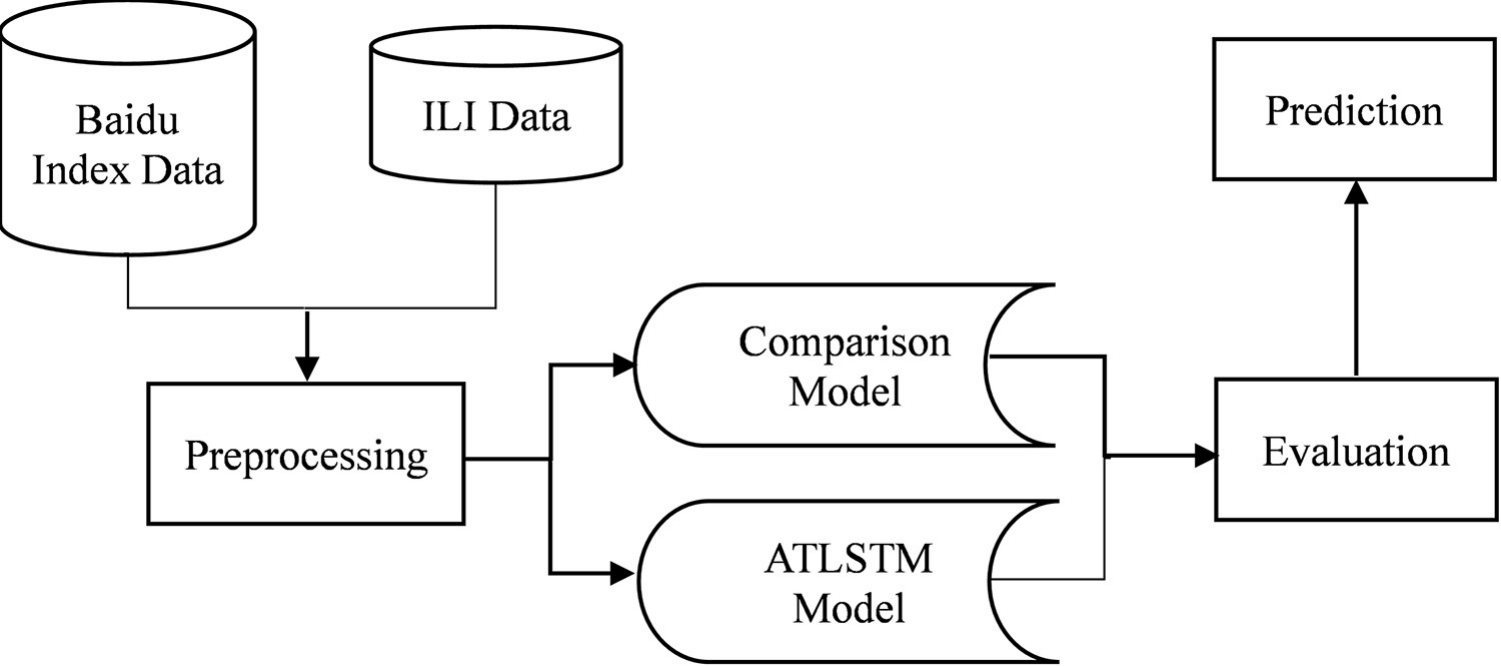
The whole working flow of our study. As shown in this figure, firstly, we give two datasets, Baidu Index data and ILI data where the former is the independent variable of the regression model and the latter is the dependent variable, for model training and testing. Secondly, through data preprocessing, we divide the two data sets into training set and test set, and retain their corresponding relationship in the time series, where Baidu index data are also standardized for doing better training. Thirdly, all models for comparison including our proposed attention-based LSTM model are trained on the same training set and tested on the same test set, so that we can get the test results of all models in different evaluation index on the test set. Fourthly, we compare these test results of all models according to the different evaluation index, so as to analyze whether the model we propose really have better fitting and prediction capabilities. Finally, if our attention-based LSTM model is proved to be better through evaluation, we will let it output and visualize the prediction results on the test set, including the correlation between the predicted values and the real values, then we can further discuss and get conclusion.

The methodology we use in this study conforms to the paradigm of data set based model comparison research. According to the guidance of this kind of methodology, we use a series of working flow to prove that our proposed model is the best in fitting and forecasting Chinese influenza like illness data based on Baidu index data compared with many models used for comparison. This working flow is shown in Fig 1, which consists of five stages: data sources, data preprocessing, models, evaluation and prediction.

In this section of Methods, in addition to stating the methodology we adopted, we will then elaborate on the data sources and models used in this study. In the part of the data sources, two types of data used in this study, influenza like illness data and Baidu Index data, are explained. When elaborating the models we used, we firstly presented the long short-term memory model and then introduced our proposed attention-based long short-term memory model. After that the models used for comparison were also introduced, and finally, we listed the evaluation index used in this study.

### Data sources

#### Influenza like illness data

Influenza like illness (ILI) data obtained by traditional public disease surveillance system is thought to be a main indicator of influenza surveillance, which are used to count the number of suspected influenza visits among all outpatients in outpost hospitals with fever accompanied by cough or sore throat. A little bit different from the cases of influenzas data, which is more directly to be used to describe the number of influenza cases collected in the hospital. in the actual influenza weekly report, the probability of ILI correlation is recorded, which is the probability of a random visit related to influenza like cases and is equal to the proportion of ILI data in all visit data, or recorded as ILI percentage (ILI%).

Chinese ILI data are provided by the ILI monitoring outpost hospitals in 31 provinces, autonomous regions and municipalities directly under the central government excluding Hong Kong, Macao and Taiwan. These data are collected in multiple copies every week and reported to the National Influenza Centre on a weekly basis [23]. The influenza centre collects, counts and issues the weekly influenza report. In this study, ILI sample data were collected from the influenza weekly report published on the website of the China National Influenza Centre. The time span was from January 1, 2011 to January 1, 2022 and the average ILI percentage of southern and northern provinces reported in the influenza weekly report was taken as the ILI data for training, which are 574 weeks in total. According to the common practice of machine learning, 90% of the training set and 10% of the test set are distinguished, 517 weeks of data from January 1, 2011 to December 6, 2020 were used as the training set of the model, and the data after December 6, 2020 were used as the test set. We train the models in the training set and test them in the test set to get results.

#### Baidu index data

Baidu Index is a data analysis platform based on Baidu’s massive Internet users’ behaviour data. Its idea is to tell users how large a keyword’s search scale is in Baidu, the change trend within a given time window and the change of relevant information and public opinion, as well as the information about the characteristics of Internet users, geographical distribution, and the search of relevant words submitted for retrieval. To assist users in data analysis, the main functional modules of Baidu Index include trend research based on single words, demand map, public opinion housekeeper, crowd portrait service, etc., as well as analysis of the overall trend, regional distribution, crowd attributes, search time characteristics of the industry, etc. [24].

Baidu search index based on a single keyword as search term takes the number of search queries of this search term as the statistical object. After specific algorithm analysis and calculation, the weighted sum of search frequency of each search term in Baidu web search is obtained, while the media search volume of Baidu is filtered and weighted. According to different sources of Baidu search, search index is divided into PC search index, mobile search index and PC + mobile search index. The Baidu Index used in this study is PC + mobile search index based on a single Chinese keyword.

GTF influenza monitoring model calculates the most common time series of 50 million Google queries in the United States in a weekly basis, and processes the weekly statistical results of queries from each state separately. This data collection process is relatively easy at the level of Google company, while the general academic research is limited by the paucity of data acquisition and processing capacity, so that it is difficult to possess and deal with such a large data set and the computing environment accordingly. Therefore, from the practical consideration, our study selects the search terms ascertained in many literature studies using Baidu Index for influenza monitoring, so as to narrow the selection range of Baidu Index data.

In the high cited literature, Wang conducted keyword primary selection by means of prior analysis and cross-correlation analysis, and the results of keyword selection were divided into prevention stage, symptom stage, treatment stage and commonly used words, totalling 79 key words [15]. Bu and others distinguished four major categories of flu keywords, which are “prevention phase”, “symptom phase”, “treatment phase”, and “commonly-used phrase” [16]. Compared with the above research, there are also some studies just using part of the search terms to establish regression model for influenza like cases modelling, and still getting the roughly same good results [13, 14, 25].

Therefore, on the basis of previous literature, our study comprehensively considers the search terms used in Baidu influenza related search, and selects the primary search terms, as shown in Table 1. From the three perspectives of commonly used words, symptom words and therapeutic words, we remove the overlapped parts of the three, and finally select 25 primary search words. The Baidu Index data used in this study is the data of the 25 primary search terms obtained from Baidu from January 1, 2015 to January 1, 2022.

**Table 1:** Search terms primarily selected in this study.

Search term type	Primarily selected search terms
Commonly used words	流感(influenza, *X*_1_)、甲流(type A influenza, *X*_2_)、H1N1(*X*_3_)、H7N9(*X*_4_)、H5N1(*X*_5_)
Symptom words	感冒(have a cold, *X*_6_)、发热(fever, *X*_7_)、呼吸道感染(respiratory tract infection, *X*_8_)、发烧(fever, *X*_9_)、高烧(high fever, *X*_10_)、鼻塞(nasal congestion, *X*_11_)、咳嗽(cough, *X*_12_)、气管炎(tracheitis, *X*_13_)、流鼻涕(rhinorrhea, *X*_14_)、咽炎(pharyngitis, *X*_15_)、咽喉痛(sore throat, *X*_16_)、咽喉炎(pharyngolaryngitis, *X*_17_)
Therapeutic words	泰诺(Tylenol, *X*_18_)、康泰克(Contac, *X*_19_)、感康(compound amantadine, *X*_20_)、白加黑(Baijiahei capsule, *X*_21_)、快克(Kuaike, *X*_22_)、达菲(Tamiflu, *X*_23_)、阿莫西林(amoxicillin, *X*_24_)、板蓝根(banlangen, *X*_25_)

Note: Our primarily selected search terms in Table 1 are labeled in this format “Chinese name (English name, variable notation)”.

### Model

#### Long short-term memory model

RNN is a kind of deep learning model, which has the ability to transfer information across time steps. The RNN model can be transformed or expanded to fit the corresponding research problems, which is thought to be a rich model family, and can be used in almost all modelling fields [26]. In practical application, in order to learn the long-term dependence and solve the problem of gradient vanishing and gradient exploding in the long-term training process of RNN model, the gating mechanism is generally introduced in the network to control the accumulated speed of information, including selectively adding new information and selectively forgetting the accumulated information before. The new module is called long short-term memory (LSTM). In this paper, the performance of basic LSTM model is also used to compare with the result of attention-based LSTM network.

The Baidu Index sequence of N retrieval words selected is used as the input data for model training or testing, and the actual data ILI time sequence is used as the dependent variable or comparison quantity for model training or testing, in which the input sequence is

$$X={\left(X_{1},X_{2},\ldots ,X_{N}\right)}^{⊺}=\left(\begin{array}{ccc} \begin{array}{cc} x_{11} & x_{12}\\ x_{21} & x_{22} \end{array} & \cdots & \begin{array}{c} x_{1T}\\ x_{2T} \end{array}\\ \vdots & \ddots & \vdots \\ \begin{array}{cc} x_{N1} & x_{N2} \end{array} & \cdots & x_{NT} \end{array}\right)\in R^{N\times T}$$ (1)

Where *X* is an n-dimensional array, which contains Baidu Index sequences of *N* search terms and of length *T*, while the output is a one-dimensional array

$$Y=(y_{1},y_{2},\ldots ,y_{T})\in R^{T}$$ (2)

Considering the design mode of LSTM algorithm and the computing power of the research, the lightweight LSTM and attention-based network that only consist of a couple of layers are studied. Let *x*_*t*_ denote the value of the input of the Baidu Index sequence at the time of *t*, *t*∈{1,2,…,*T*}, *h*_*t*_∈*R*^*M*^ is the hidden state of the basic *M*-unit LSTM cell which has a memory cell unit with the state *s*_*t*_∈*R*^*M*^ at time *t*. The forget gate, input gate and the output gate of this LSTM cell are noted as *f*_*t*_, *i*_*t*_, and *o*_*t*_, so the update process can be expressed as

$$f_{t}=\sigma (W_{f}[h_{t-1};x_{t}]+b_{f})$$ (3)

$$i_{t}=\sigma (W_{i}[h_{t-1};x_{t}]+b_{i})$$ (4)

$$o_{t}=\sigma (W_{o}[h_{t-1};x_{t}]+b_{o})$$ (5)

$$s_{t}=f_{t}⊙s_{t-1}+i_{t}⊙tanh(W_{s}[h_{t-1};x_{t}]+b_{s})$$ (6)

$$h_{t}=o_{t}⊙tanh(s_{t})$$ (7)

where [*h*_*t*−1_; *x*_*t*_]∈*R*^*M*+*N*^ is the concatenation of the previous hidden state *h*_*t*−1_ and the current input *x*_*t*_, *σ* and ⊙ are logistic sigmoid function and element-wise multiplication respectively, *W*_*f*_, *W*_*i*_, *W*_*o*_, *W*_*s*_∈*R*^*M*×(*M*+*N*)^ are weights and *b*_*f*_, *b*_*i*_, *b*_*o*_, *b*_*s*_∈*R*^*M*^ are bias to learn. The whole update process of this LSTM cell can be summarized as

$$h_{t}=LSTM(h_{t-1},x_{t})$$ (8)

When training this cell, we propose the minibatch stochastic gradient descent (SGD) together with the Adam optimizer methods, which are also implemented to train the attention-based LSTM model. There are also fully connected layers added after this LSTM cell to form the entire model, which also used in the attention-based model, and they are both learned by same standard back propagation with mean squared error function as the objective function that described below.

#### Attention-based LSTM model

Inspired by the theory that the human attention system can select elementary stimulus features in the early stages of processing, the attention mechanism is proposed to adaptively select the relevant driving series, which calculates the attention weights corresponding to each sequence of input data, and improves the performance of time series prediction [27].

The attention-based LSTM network uses the hidden state $h_{t}^{\prime }$ and the memory state $s_{t}^{\prime }$ of the *M*-unit LSTM cell to capture the attention weights of each input sequence *X* = (*X*_1_,*X*_2_,…,*X*_*N*_)^*T*^. We use $X_{K}=(x_{K,1},x_{K,2},\ldots ,x_{K,T})\in R^{T},K\in \{1,2,\ldots ,N\}$, to denote Baidu Index sequences of the K^th^ search term at the time of *t*, *V*_*e*_∈*R*^*T*^, *W*_*e*_∈*R*^*T*×2*M*^, and *U*_*e*_∈*R*^*T*×*T*^ are weights to learn, then a deterministic attention mechanism is computed as

$$e_{K,t}=V_{e}^{⊺}tanh(W_{e}[h_{t-1}^{\prime };s_{t-1}^{\prime }]+U_{e}X_{K})$$ (9)

By using Softmax function on each *e*_*K*,*t*_, there are attention weights

$$\alpha_{K,t}=\frac{exp(e_{K,t})}{\sum_{i=1}^{N}exp(e_{i,t})}$$ (10)

Then we can adaptively extract the original sequence *x*_*t*_ into

$$\tilde{x_{t}}={\left(\alpha_{1,t}x_{1,t},\alpha_{2,t}x_{2,t},\ldots ,\alpha_{N,t}x_{N,t}\right)}^{⊺}$$ (11)

Let $f_{t}^{\prime },i_{t}^{\prime }$, and $o_{t}^{\prime }$ denote the forget gate, input gate and the output gate of LSTM cell, $W_{f}^{\prime },W_{i}^{\prime },W_{o}^{\prime },W_{s}^{\prime }\in R^{M\times (M+N)}$ are weights and $b_{f}^{\prime },b_{i}^{\prime },b_{o}^{\prime },b_{s}^{\prime }\in R^{M}$ are bias, we have the update process

$$f_{t}^{\prime }=\sigma (W_{f}^{\prime }[h_{t-1}^{\prime };\tilde{x_{t}}]+b_{f}^{\prime })$$ (12)

$$i_{t}^{\prime }=\sigma (W_{i}^{\prime }[h_{t-1}^{\prime };\tilde{x_{t}}]+b_{i}^{\prime })$$ (13)

$$o_{t}^{\prime }=\sigma (W_{o}^{\prime }[h_{t-1}^{\prime };\tilde{x_{t}}]+b_{o}^{\prime })$$ (14)

$$s_{t}^{\prime }=f_{t}^{\prime }⊙s_{t-1}^{\prime }+i_{t}^{\prime }⊙tanh(W_{s}^{\prime }[h_{t-1}^{\prime };\tilde{x_{t}}]+b_{s}^{\prime })$$ (15)

$$h_{t}^{\prime }=o_{t}^{\prime }⊙tanh(s_{t}^{\prime })$$ (16)

The whole update process of this attention-based LSTM cell can be summarized as

$$h_{t}^{\prime }=Attention-based\;LSTM(h_{t-1}^{\prime },\tilde{x_{t}})$$ (17)

In order to gain a better training result for this model, we also add fully connected layers by concatenating the LSTM output $h_{t}^{\prime }$ with the previous target sequence value *y*_*t*−1_, if this target value is available in actual practice, so there is

$${\hat{y}}_{t}=V_{y}^{⊺}(W_{c}[h_{t}^{\prime };y_{t-1}]+b_{c})+b_{y}$$ (18)

where $[h_{t}^{\prime };y_{t-1}]\in R^{M+1}$ is the concatenation of $h_{t}^{\prime }$ and *y*_*t*−1_, which mapped by *W*_*c*_∈*R*^*M*×(*M*+1)^ and *b*_*c*_∈*R*^*M*^, while *V*_*y*_∈*R*^*M*^ and *b*_*y*_∈*R* are parameters of the final linear function to produce the model outputs. When training this cell, we use standard back propagation with mean squared error function as the objective function, considering $Y_{K}=(y_{K,1},y_{K,2},\ldots ,y_{K,T})\in R^{T},K\in \{1,2,\ldots ,N\}$, there is

$$O(y_{T},{\hat{y}}_{T})=\frac{1}{N}\sum_{i=1}^{N}{\left({\hat{y}}_{i,T}-y_{i,T}\right)}^{2}$$ (19)

**Fig 2:**
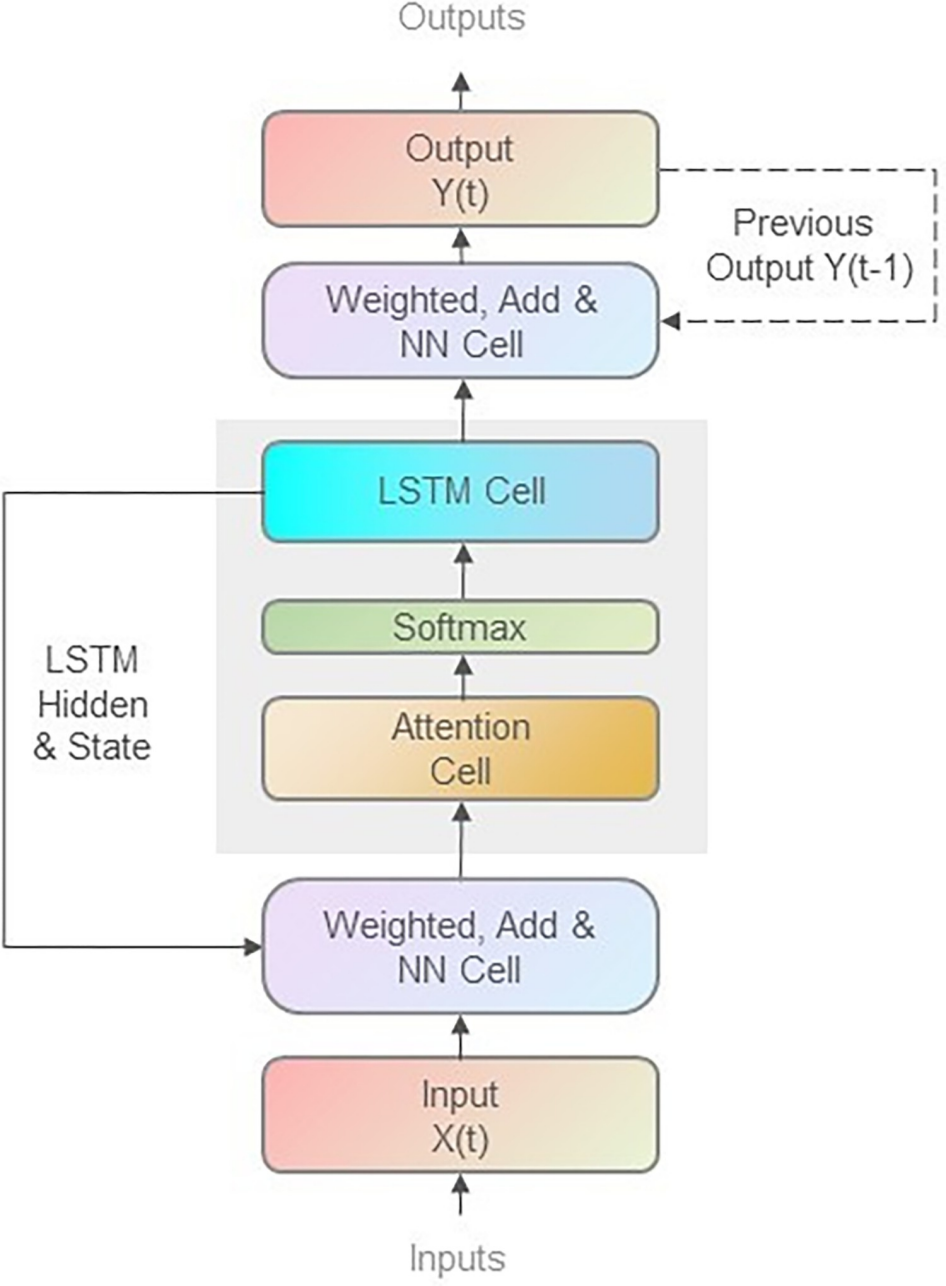
The structure diagram of our proposed attention-based LSTM model. The main structure of our proposed attention-based LSTM model is composed of Input, NN Cell, Attention Cell, Softmax, LSTM Cell, and Output modules. Corresponding to the mathematical expressions above, firstly the Input sequences X(t) will be weighted and add together with the weighted LSTM output Hidden and State data, then go through the NN Cell; secondly the data will flow to Attention Cell to generate the attention weighted data and then go into Softmax layer; thirdly the LSTM Cell will operate on the input data and output Hidden and State data from two directions, one is to output to the previous NN layer to operate with the data of the next sequence, and the other is output to the later NN layer for operating the model output Y(t). In order to gain a better training result for this model, we can also add a step that concatenating the previous output sequence data Y(t-1) with the LSTM Cell output to get the latest output sequence Y(t) in the last NN layer.

To establish a real experimental model, we use the structure of our proposed attention-based LSTM model shown in Fig 2 in this study. When coding to realize those models, the TensorFlow framework with Keras module is used to implement the minibatch stochastic gradient descent (SGD) and the Adam optimizer method to train them.

Before training and comparing with other models, as for our proposed deep learning model, two important training issues need to be considered, which are overfitting issues and underfitting issues. Here, overfitting is reflected in the weak generalization ability of the training model and poor prediction performance on the extensive test data set, and underfitting is reflected in the poor convergence of the training model during training and poor effect both on the training data set and test data set.

For dealing with the overfitting issues, several directions have been considered. Firstly, the data we use are sufficient and comprehensive enough, where 574 time series data with one ILI sequence and twenty-five Baidu Index sequences are used to feed our model, which improves the robustness of our training effect from the data level. Secondly, our proposed attention-based LSTM structure is a lightweight and appropriate model to solve our target problem, we avoid the overfitting possibility came from model complexity. Thirdly, we use early stopping technics to truncate the number of iterations when the optimization degree of training gradually falls into a small area, that is, stop iteration before the model converges too much to the training dataset to prevent overfitting.

Underfitting problem will not occur in our training process, on one hand, we carefully set the number of iterations of our model learning curve where we make sure that it represents sufficient convergence characteristics; on the other hand, our tools and components used are widely recognized by the industry, in where the network layers and model parameters constructed are robust enough to ensure that the fitting process is simple and effective. Additionally, although many deep learning methods have used dropout component, which refers to randomly "deleting" a part of hidden units (neurons) with a certain probability during training, the improper use of this dropout function will lead to new underfitting problems, hence this method is not used in our work since our model settings are just simple and efficient enough.

#### Comparison model

Theoretically, every research in the influenza surveillance field has its own characteristic, but we only choose their algorithms for comparison. In our opinion, the rationale of this kind of research methodology can be explained by the following reasons. Firstly, to see whether the model we provide has relatively advantages, we need to compare it with some other models. Secondly, there are many models that can be compared while it is impossible to compare them all, hence for choosing those models, two factors are mainly considered in the selection: the one is that we choose some models not only suitable in our research background, but also commonly used in the fitting and prediction of infectious diseases; the other is that we also choose two basic deep learning models without further deep learning design, which are LSTM and RNN, so as to see the advantages of deep learning mechanism such as our attention-based model. Thirdly, for many studies, the data and programming methods they used are difficult to reproduce, and the evaluation methods and indicators used in some studies are also different from ours. Therefore, we can only seek to compare those algorithms, which can be realized and obtain results under the data sets and evaluation index of our working flow, with our models in this study.

Therefore, for comparing the performance of our models proposed above, we then set up models that selected from other researches in the influenza surveillance field as the control group. Firstly we consider the autoregressive moving average model ARMA(p, q) and the autoregressive integrated moving average model ARIMA(p, d, q) to forecast the trend of ILI influenza data, where p is the number of autoregressive (AR) terms, and q is the order of the non-seasonal moving average (MA) lags, and d is the number of non-seasonal differences. Respectively, the ARMA(p, q) model can be formulated as

$$y_{t}=\omega_{0}+\sum_{i=1}^{p}\varphi_{i}y_{t-i}+\sum_{j=1}^{q}\omega_{j}\varepsilon_{t-j}+\varepsilon_{t}$$ (20)

and ARIMA(p, d, q) model is

$$w_{t}={∆}^{d}y_{t}={\left(1-L\right)}^{d}y_{t}$$ (21)

$$w_{t}=\omega_{0}+\sum_{i=1}^{p}\varphi_{i}w_{t-i}+\sum_{j=1}^{q}\omega_{j}\varepsilon_{t-j}+\varepsilon_{t}$$ (22)

where *y*_*t*_ is the number of ILI influenza data at time t and *ε*_*t*_ is white noise random error, Δ^*d*^ = (1−*L*)^*d*^ are *d*-th order differential operators, *φ*_*i*_ (*i* = 1,2,…,*p*) and *ω*_*j*_ (*j* = 1,2,…,*q*) are parameters to be estimated via maximum likelihood estimation. Moreover, the corrected Akaike Information Criterion (AIC) test is set to selected the parameters p, q and d from the possible model candidates.

The classic linear regression model and multi-layer perceptron model are widely implemented in the field of influenza monitoring and modelling. Among them, multiple linear regression (MLR) aims to explore the linear correlation between multiple independent variables and a dependent variable. For n-dimensional Baidu Index sequence *X*(*t*) and ILI influenza data *Y*(*t*), there are

$$Y=\alpha_{0}+\alpha X+\varepsilon$$ (23)

$$\alpha ={\left(\alpha_{1},\alpha_{2},\ldots ,\alpha_{N}\right)}^{T}$$ (24)

In this expression, *ε* is the error term that has a normal distribution, the regression model determines the parameters under the least square method, which can be formulated as

$$argmin\;J_{MLR}(\alpha )=argmin\frac{1}{N}{\left\|Y-\alpha X\right\|}^{2}$$ (25)

In many cases, the ridge regression (RR) and least absolute shrinkage and selection operator regression (LASSO regression, LSR) are also used as the improvement and supplement of MLR model. By conducting L1 regularization method, the least square method of LSR model can be formulated as

$$argmin\;J_{LSR}(\alpha )=argmin\frac{1}{N}{\left\|Y-\alpha X\right\|}^{2}+\lambda \|\alpha \|$$ (26)

while by conducting L2 regularization method, the least square method of RR model can be formulated as

$$argmin\;J_{RR}(\alpha )=argmin\frac{1}{N}{\left\|Y-\alpha X\right\|}^{2}+\lambda {\left\|\alpha \right\|}^{2}$$ (27)

A basic RNN algorithm is also designed, the input layer, the hidden layer and the output layer contain I, H and K neurons, and the forward propagation algorithm for the hidden layer RNN can be expressed as

$$a_{h}^{t}=\sum_{i=1}^{I}w_{ih}x_{i}^{t}+\sum_{j=1}^{H}w_{jh}b_{j}^{t-1}$$ (28)

$$b_{h}^{t}=\theta_{h}(a_{h}^{t})$$ (29)

$$a_{k}^{t}=\sum_{j=1}^{H}w_{jk}b_{j}^{t}$$ (30)

where $x_{i}^{t}$ is the value of the i-th input of the sequence at the time of *t*, *w*_*ij*_ is the connection weight of neuron *i* and *j* at the time of *t*, $a_{j}^{t}$ and $b_{j}^{t}$ are the value of neuron *j* at the time of *t*, and *θ*_*h*_ is the activation function corresponding to neuron *h*. Similarly, the original version of deep neural network, multi-layer perceptron (MLP) is also compared, its input layer and hidden layer contain I and H neurons, whose forward propagation algorithm is expressed as

$$a_{h}^{t}=\sum_{i=1}^{I}w_{ih}x_{i}^{t}$$ (31)

$$b_{h}^{t}=\theta_{h}(a_{h}^{t})$$ (32)

$$a_{k}^{t}=\sum_{j=1}^{H}w_{jk}b_{j}^{t}$$ (33)

The loss function is also defined by the chain rule, and the calculation of the loss function is only affected by the current time. The weight updating algorithm of multi-layer perceptron is similar to RNN.

Finally, the support vector regression (SVR) model evolved from the support vector machine (SVM) model is one of the widely used machine learning models, which can also be used in the research of influenza monitoring. For the data set used in the research, $D≔{\left\{\left(X_{t},Y_{t}\right)\right\}}_{t=1}^{T}(X_{t}\in R^{N},Y_{t}\in R)$, the SVR model maps the input space *R*^*N*^ into high dimensional space by using nonlinear function Φ(*X*), and uses the linear function to fit the data set ${\left\{\left(\Phi (X_{t}),Y_{t}\right)\right\}}_{t=1}^{T}$ in the high dimensional feature space. There are

$$Y=f(X)=W^{T}\Phi (X)+b$$ (34)

The objective function of the program is

$$R(w)=\frac{1}{2}w^{T}w+\lambda \sum_{n=1}^{N}{\left|Y_{n}-(W^{T}\Phi (X)+b)\right|}_{\varepsilon }$$ (35)

Therefore, the parameters of the model can be obtained by Lagrange multiplier method, and the kernel function technique is often used in the specific solution process. The support vector kernel function applied in this study is Gaussian kernel function (RBF kernel). In Baidu Index fitting model, this study uses the same training data set to train those six models mentioned above.

#### Evaluation index

For given Baidu Index and ILI data, the RNN model, LSTM model, attention-based LSTM model, autoregressive moving average, autoregressive integrated moving average, multiple linear regression, ridge regression, LASSO regression, multi-layer perceptron and support vector regression trained in model training steps are examined at the test set respectively, so that there is a total of 10 examining results. Considering the background of the model prediction problem, the examining method is to examine the test sequence calculated by each model on the test set, and to carry out mean absolute error (MAE), mean absolute percentage error (MAPE), symmetric mean absolute percentage error (SMAPE), mean square error (MSE), root mean square error (RMSE), R-square value and index of agreement (IA) test on the ILI test set of influenza like cases, and to give the ranking of the model on these seven indexes, among which

$$MAE=\frac{1}{T}\sum_{t=1}^{T}\left|y_{t}-{\hat{y}}_{t}\right|$$ (36)

$$MAPE=\frac{100\%}{T}\sum_{t=1}^{T}\left|\frac{y_{t}-{\hat{y}}_{t}}{y_{t}}\right|$$ (37)

$$SMAPE=\frac{100\%}{T}\sum_{t=1}^{T}\frac{2|y_{t}-{\hat{y}}_{t}|}{\left(|y_{t}|+|{\hat{y}}_{t}|\right)}$$ (38)

$$MSE=\frac{1}{T}\sum_{t=1}^{T}{\left(y_{t}-{\hat{y}}_{t}\right)}^{2}$$ (39)

$$RMSE=\sqrt{MSE}$$ (40)

The size of the error between the predicted sequence and the actual sequence is described, where ${\hat{y}}_{t}$ is the term in the predicted sequence and $\bar{y}$ is the mean of all of the value *y*_*n*_, and

$$R-square=1-\frac{\sum_{t=1}^{T}{\left(y_{t}-{\hat{y}}_{t}\right)}^{2}}{\sum_{t=1}^{T}{\left(y_{t}-\bar{y}\right)}^{2}}$$ (41)

In this paper, we characterize the consistency of distribution between the predicted sequence and the real sequence, where *P*_*t*_ and *O*_*t*_ are the terms in the predicted sequence and the real sequence respectively, and $\bar{O}$ represents the mean value of the real sequence. There is index of agreement computed as

$$IA=1-\frac{\sum_{t=1}^{T}{\left(P_{t}-O_{t}\right)}^{2}}{\sum_{t=1}^{T}{\left(|P_{t}-\bar{O}|+|O_{t}-\bar{O}|\right)}^{2}}$$ (42)

This formula reflects the covariant relationship between the predicted value and the real value and the "real mean". The closer the IA value is to 1, the more similar the distribution is, the more reliable the fitting result is [28].

The results of these 70 experiments on these seven indicators were ranked respectively to determine which model performed better, and the best model was selected from all the test results of these two indicators, through which the follow-up influenza trend was simulated, and the differences of simulation results were simply compared.

## Results

### Training and comparison results

For the given N = 25 search terms, a group of models are trained. The training model is listed in Table 2. In this study, MLR, MLP and SVR model programs are written using Python based skeleton machine learning framework, and RNN research model programs are written using Python based Google TensorFlow deep learning framework.

**Table 2:** The training models and their abbreviations.

Type of models	Description	Abbreviation
Our models	Attention-based LSTM network	ATLSTM
	Long-short term memory network	LSTM
	Recurrent neural network	RNN
Models for comparison	Autoregressive moving average	ARMA
	Autoregressive integrated moving average	ARIMA
	Multiple linear regression model	MLR
	Ridge regression	RR
	LASSO regression	LSR
	Four-layer fully connected multi-layer perceptron network	MLP
	Support vector regression	SVR

In the process of model training, the number of neurons in each hidden layer of RNN, LSTM and MLP model is set to 32, the time step of RNN and RNN-LSTM model is set to 3, and the data shift of training sequence is 1 step. In the model training phase, we use data from the 1st week of 2011 to the 50th week of 2020 to train those models. In terms of the training process, the training epochs of the RNN-kind models is 64, and all models have achieved good convergence effect.

As the test set to evaluate all the models trained on the training set, model regression prediction step is carried out on the test set data of models obtained through training from the last 57^th^ weeks, which is the 49^th^ week of 2020 to the 52^th^ week of 2021, and 10 groups of prediction series are obtained. The comparison results of these prediction series and actual series on MAE and IA indexes are shown in Table 3.

**Table 3:** Test results of the training models.

Model	MAE	IA	MAPE	SMAPE	MSE	RMSE	R-square
ARMA	0.423	0.379	0.162	0.145	0.289	0.538	-0.272
ARIMA	0.714	0.342	0.234	0.206	1.406	1.186	-5.188
MLR	0.242	0.816	0.086	0.085	0.104	0.323	0.542
RR	0.274	0.749	0.096	0.096	0.122	0.350	0.461
LSR	0.246	0.800	0.085	0.086	0.108	0.329	0.525
MLP	0.511	0.616	0.183	0.165	0.382	0.618	-0.680
SVR	0.352	0.748	0.127	0.121	0.174	0.417	0.236
RNN	0.284	0.750	0.101	0.100	0.134	0.366	0.409
LSTM	0.239	0.807	0.088	0.084	0.103	0.321	0.546
ATLSTM	**0.189**	**0.929**	**0.066**	**0.066**	**0.056**	**0.237**	**0.752**

As shown in Table 3, it can be found from the error index of prediction results that the MAE, MAPE, SMAPE, MSE and RMSE between attention-based LSTM model with the actual ILI data is the minimum among them, followed by LSTM model and MLR model. On the whole, RNN-kind models perform well in those error index, and it’s noteworthy that in a long time span with limited ILI fluctuation range, the traditional regression tools like RLR, RR and LSR model also have a good performance. The SVR model is acceptably good while the MLP, ARMA and ARIMA model have achieved big prediction error in different index.

**Fig 3:**
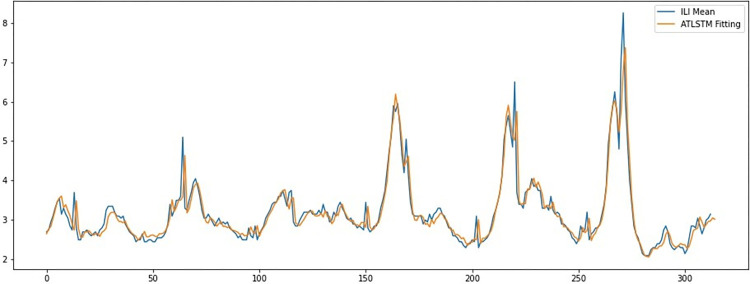
Fitting results of the attention-based LSTM model. The abscissa of this line chart is the time span (in week unit, containing the last 315 weeks of the training set, from the 49^th^ week of 2014 (December 1^st^ to December 7^th^), to the 50^th^ week of 2020 (December 7^th^ to December 13^th^)), and the ordinate of this chart is the numerical value of ILI. In this chat the real numerical value of ILI mean data through the time span is shown as the blue line, and the algorithm return ILI value by using ATLSTM model in fitting the training dataset in the same time span is shown as the brown line. In this line chart, there is a high degree of fitness between the blue line and the brown line, which reveals the characteristics of the real ILI data are well captured by the ATLSTM by training based on the Baidu index data.

From the IA and R-square indicators of the prediction results shown in Table 3, it can be found that the distribution consistency of attention-based LSTM model is the closest to the actual ILI data, followed by MLR model and LSTM model. On the whole, RNN-kind models performs very well in the consistency index test, which reveals those models have the ability to master more details in the learning of training data. Fig 3 shows the fitting results of the attention-based LSTM model in the last 315 training sequences with the ILI mean data.

It can also be found in Table 3 that the performance of MLR model on MAE index and IA consistency index is better than MLP and SVR model. Among them, the fitting performance of MLR on influenza monitoring based on Baidu Index is very general, which is consistent with the expectation of the traditional machine learning field in the fitting performance of these three types of models. Before the rise of deep learning model, support vector machine usually has better model effect than multi-layer perceptron, which has been widely acknowledged.

We also simply compare our results with the new results of deep learning models done by three studies in the field of influenza surveillance. All of these models only consider fitting and predicting the ILI data by mining the characteristics of the ILI time series data itself, and none of them use Baidu Index as the input for predicting the ILI data, which are different from our model. Moreover, the selection of evaluation index in these studies is not as comprehensive as ours, and considering the feasibility of reproducing them, we only select the existing results in these studies for comparison.

Specifically, The MAE and RMSE results of our proposed ATLSTM model are 0.189 and 0.237 respectively, while those results of the GA-LSTM model given by Kara [19] are 3173 and 5166, and those results of the GA-ConvLSTM-CNN given by Kara [20] are 186.83 and 297.74. It is worth noting that the latter two studies did not standardize their error index data, so although our results are looking better, it is difficult for us to truly compare this result with ours. The R-square results of GA-LSTM and GA-ConvLSTM-CNN is 0.892 and 0.837 respectively, while the R-square results of our model is 0.752. For the study given by Zhu et al. [21], they report the MAPE result of their proposed Att-MCLSTM model is 0.086, while the MAPE result of ours is 0.066, which shows our ATLSTM model may do better in this scenario.

### Future prediction

**Fig 4:**
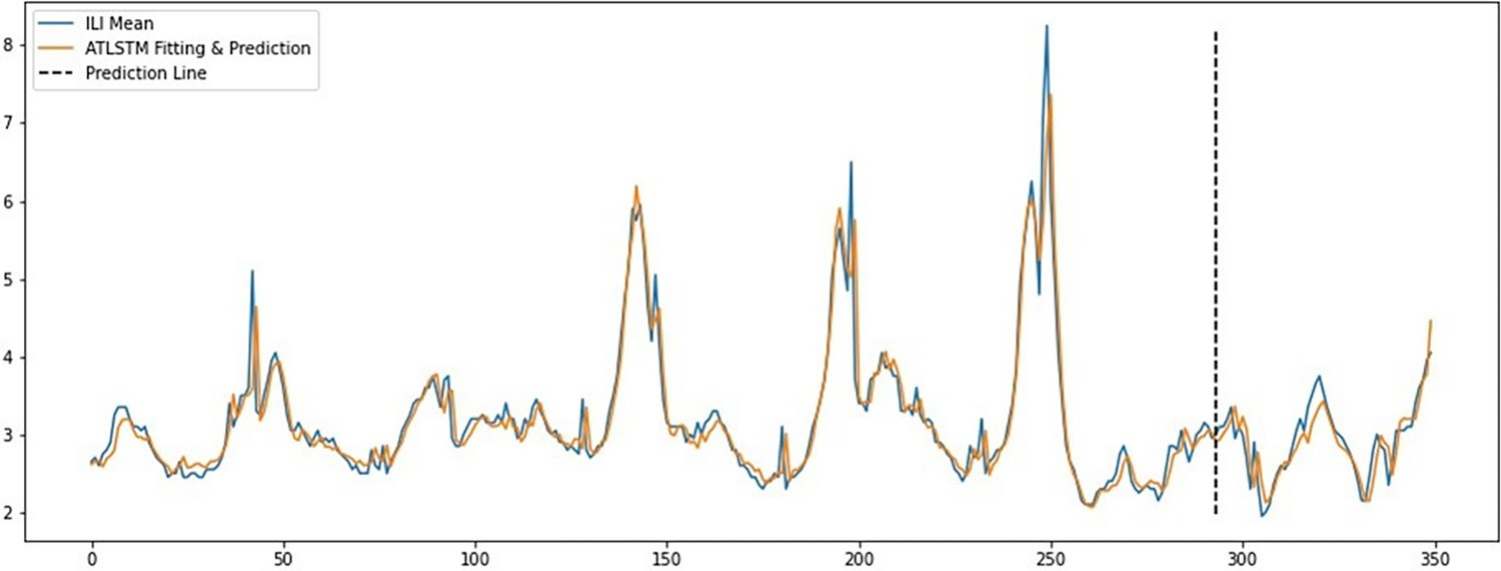
Prediction results of the attention-based LSTM model. The abscissa of this line chart is the time span (in week unit, containing the last 350 weeks of the whole dataset, from the 52^th^ week of 2015 (December 21^th^ to December 27^th^), to the 52^th^ week of 2021 (December 27^th^ 2021 to January 2^nd^ 2022)), and the ordinate of this chart is the numerical value of ILI. Since we use the last 57 week of our whole data as the test set, in this chat we draw a dotted line named prediction line to distinguish the time span between training data and test set, which is, the 49^th^ week of 2020 (November 30^th^ to December 6^th^) to the 52^th^ week of 2021. Same as the Fig 2, in this chart, the real numerical value of ILI mean data through the time span is shown as the blue line, and the algorithm return ILI value by using ATLSTM model in fitting and prediction on the training set and test set respectively in the same time span is shown as the brown line. In this line chart, we can also find a high degree of fitness between the blue line and the brown line, which reveals the characteristics of the real ILI data are well remembered by the ATLSTM by training based on the Baidu index data so it can return a good prediction.

The model with the best performance in those evaluation indexes, ATLSTM, which is attention-based LSTM model, is used to prediction the ILI mean values by using the latest Baidu Index test data and the corresponding ILI mean data collected after December 2020. Fig 4 shows the prediction results of this model as of the completion to the real ILI mean data, where the Prediction Line is used as the segmentation line of fitting data and prediction data.

**Fig 5:**
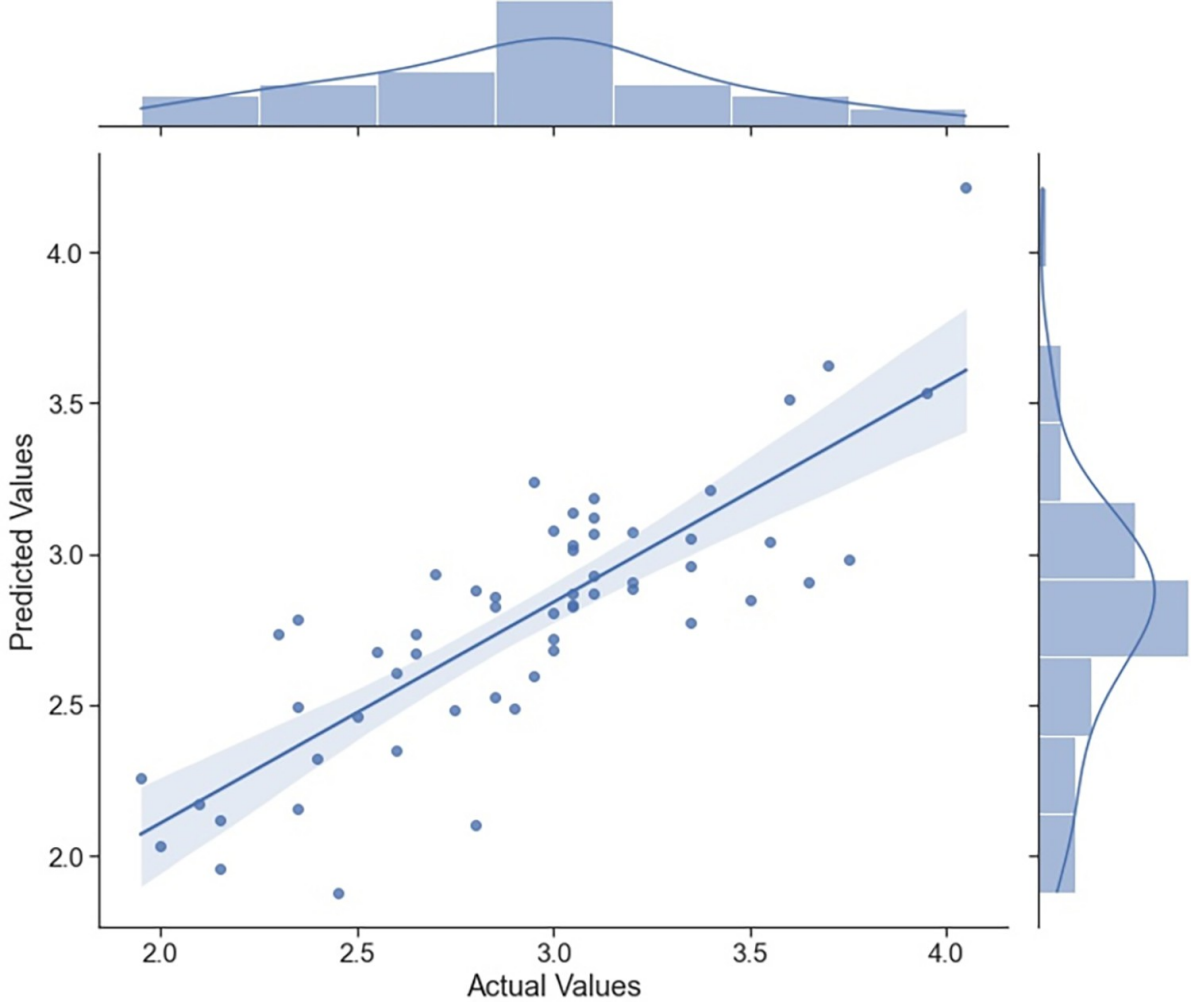
Correlation of the predicted values of the attention-based LSTM model and the actual values. The data presented in this figure are all time series data in the same time interval, which is, the time interval we used for model testing from 49^th^ week of 2020 (November 30^th^ to December 6^th^) to the 52th week of 2021. The abscissa of this scatter plot in the main figure is the actual ILI values and the ordinate of this chart is the predicted values of our proposed ATLSTM model. The regression line of those scatter points is also given in the main figure, where the shadow around the line represents the confidence interval. The two auxiliary figures on the top and on the right are the distribution plots of actual values and predicted values respectively. As a supplement to the known results that R-square of our proposed ATLSTM model is 0.752, in this line figure, we can also find a high degree of correlation between predicted values and actual values, which show the ATLSTM model can return a good prediction.

As shown in Fig 4, on the left of the vertical straight line, prediction Line, is the fitting results and prediction is on the right. The fitting and prediction results of the model and ILI mean data are visualized in the figure, which shows that most of the details of the actual ILI value are grasped and remembered by the ATLSTM model. Moreover, Fig 5 shows the correlation of the predicted values of the ATLSTM model and the actual ILI values, which also provides evidence of high prediction level of the ATLSTM model. From the prediction results of Baidu Index model based on the last prediction task, we can find the attention-based LSTM model can correctly track the trend of influenza epidemic in China.

## Discussion

Timely identification and early warning of influenza epidemic situation has become one of the most important issues in the practice of influenza prevention. The influenza monitoring system based on Baidu Index can be ahead of the traditional influenza weekly report in national influenza centre such as CDC or CNIC for 1–2 weeks by real-time retrieving and processing data. This is the biggest advantage of influenza monitoring based on search engine, which can further improve the accuracy of the monitoring system. On this basis, more accurate fitting and prediction of the development trend of influenza should be an important development direction of influenza monitoring system. In our study, the advanced deep learning algorithm is used to combine Baidu Index with RNN-kind models for the first time, which opens up a new research idea for influenza surveillance research based on search engine data from the method level. Moreover, our proposed model is almost no different from other simple models in data usage and operation efficiency, but has achieved better results.

Compared with the previous influenza monitoring models introduced above, our proposed attention-based LSTM structure trained in the research experiment reveals its capacity to capture the more subtle features in the mapping process from the Baidu Index data to the actual influenza survey data. Through fitting the characteristics of sequence memory, long-term memory and sequence attention of the model itself, our structure is done to reach the best accuracy and consistency results in the influenza surveillance field. Although the attention-based LSTM model has been used in the field of financial marketing [29], machine translation [30], sentiment classification [31] and even in the application of fitting illness cases [32], our contribution is first introducing this method to engage with the Baidu Index in the influenza surveillance field, which has been proved to be a very intuitive but effective method.

In the condition of fitting and prediction based on internet retrieving, it should be noticed that the use of search engine data for influenza monitoring, as a matter of fact, can only guarantee the modelling results has a certain relevance with the actual influenza epidemic situation if the size of the search population is large enough. When using search engine data for influenza monitoring, the query data obtained by search engine is not only the query submitted by users with suspected flu symptoms, and although it is relevant in historical data, for example, a special event such as a flu drug developer winning the Nobel prize may cause sudden fluctuation to search engine, thus there are false warnings in the influenza monitoring system [18]. In addition to model improvement, data depolarization technics will also be a development direction, which will be the considerable components when using Baidu Index for influenza monitoring.

There are also other diseases being modelled by using search engine data, such as H7N9, dengue [33], gonorrhoea [34], brucellosis [35], AIDS [17] and Covid 19 [36–38]. In these studies, most of their models are based on the time series data and just traditional and simple, which can be improved and tested by our attention-based LSTM structure. For more widely application, from a higher perspective, the generalized disease monitoring system based on search engine data and actual ILI data can be further integrated, in which different model methods can be compared and produce the best prediction results, including those advanced models.

With the increasingly popularity of deep learning algorithm operation ability, more complex and precise modelling methods will continue to innovate and develop in various fields. For the influenza monitoring problem, moreover, in achieving better data access efficiency and processing, not only the more powerful algorithms or more effective systems will be applied, but more comprehensive and diversified external data will also be innovated to serve our public health services. There are also some works focusing on retrieving data from different regions and different search engines [39], News data [40], human gene database [41], spatiotemporal feature data [42] or data from social networking platform such as Weibo [43], Instagram [44] or Twitter [45], which further expand the scope of support availability in the field of influenza surveillance. Therefore, in the future, a greater degree of big data enabling disease detection and prevention will also be the trend that researchers need to focus on, where the work of diversified external data collection and application will also be considered as an important contribution.

## Supporting information

S1 Data (RAR)
